# 2,3-Dimeth­oxy­benzaldehyde azine

**DOI:** 10.1107/S1600536811003217

**Published:** 2011-01-29

**Authors:** Qamar Ali, Itrat Anis, M. Raza Shah, Seik Weng Ng

**Affiliations:** a H.E.J. Research Institute of Chemistry, International Center for Chemical and Biological Sciences, University of Karachi, Karachi 7527, Pakistan; b Department of Chemistry, University of Malaya, 50603 Kuala Lumpur, Malaysia

## Abstract

There are one-and-a-half independent mol­ecules in the asymmetric unit of the title compound, C_18_H_20_N_2_O_4_. One mol­ecule is centrosymmetric with the mid-point of the N—N bond located on a center of inversion. In the other, which lies on a general position, the benzene rings are aligned at 21.6 (1)°. Weak inter­molecular C—H⋯O hydrogen bonding is present in the crystal strcture.

## Related literature

For the structure of 2,4-dibenzaldehyde azine, see: Islam *et al.* (2009 ▶).
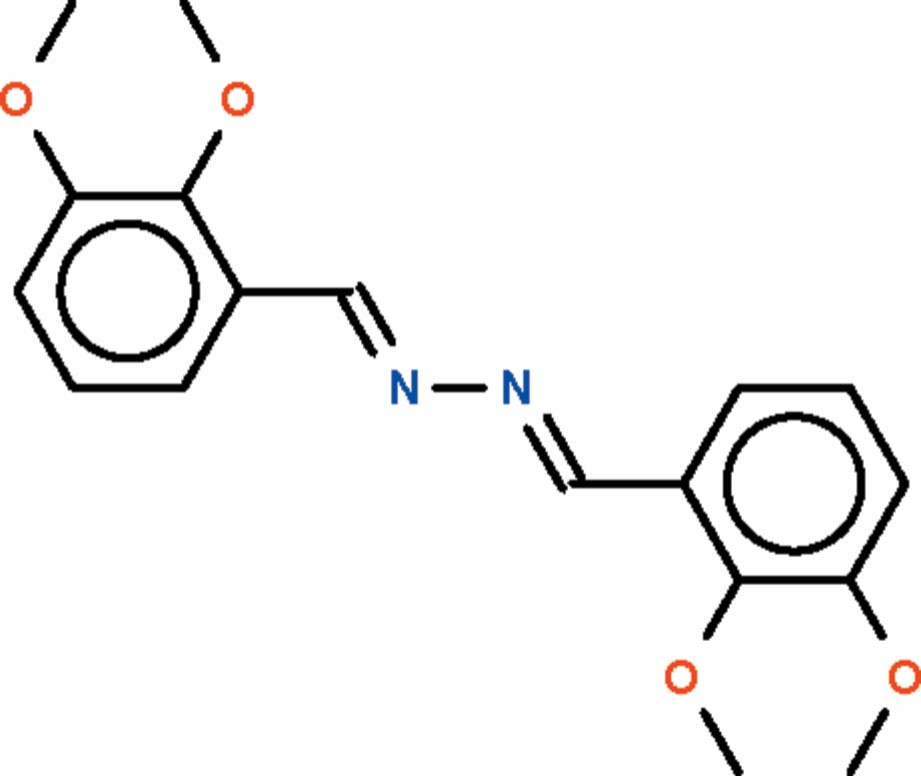


## Experimental

### 

#### Crystal data

C_18_H_20_N_2_O_4_
*M* *_r_* = 328.36
Monoclinic, 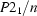

*a* = 8.0294 (2) Å
*b* = 17.9415 (5) Å
*c* = 17.5258 (4) Å
β = 96.660 (2)°
*V* = 2507.72 (11) Å^3^
*Z* = 6
Mo *K*α radiation
μ = 0.09 mm^−1^
*T* = 100 K
0.30 × 0.25 × 0.20 mm

#### Data collection

Agilent SuperNova Dual diffractometer with an Atlas detector
Absorption correction: multi-scan (*CrysAlis PRO*; Agilent, 2010) ▶ *T*_min_ = 0.770, *T*_max_ = 1.000
12722 measured reflections
5600 independent reflections
4465 reflections with *I* > 2σ(*I*)
*R*_int_ = 0.029

#### Refinement

*R*[*F* ^2^ > 2σ(*F* ^2^)] = 0.046
*wR*(*F* ^2^) = 0.125
*S* = 1.03
5600 reflections
325 parameters
H-atom parameters constrained
Δρ_max_ = 0.31 e Å^−3^
Δρ_min_ = −0.22 e Å^−3^

### 

Data collection: *CrysAlis PRO* (Agilent, 2010 ▶); cell refinement: *CrysAlis PRO*; data reduction: *CrysAlis PRO*; program(s) used to solve structure: *SHELXS97* (Sheldrick, 2008 ▶); program(s) used to refine structure: *SHELXL97* (Sheldrick, 2008 ▶); molecular graphics: *X-SEED* (Barbour, 2001 ▶); software used to prepare material for publication: *publCIF* (Westrip, 2010 ▶).

## Supplementary Material

Crystal structure: contains datablocks global, I. DOI: 10.1107/S1600536811003217/xu5148sup1.cif

Structure factors: contains datablocks I. DOI: 10.1107/S1600536811003217/xu5148Isup2.hkl

Additional supplementary materials:  crystallographic information; 3D view; checkCIF report

## Figures and Tables

**Table 1:** Hydrogen-bond geometry (Å, °)

*D*—H⋯*A*	*D*—H	H⋯*A*	*D*⋯*A*	*D*—H⋯*A*
C9—H9*B*⋯O3^i^	0.98	2.45	3.288 (2)	143
C14—H14*A*⋯O5^ii^	0.95	2.37	3.2890 (19)	161
C18—H18*C*⋯O6^ii^	0.98	2.54	3.5229 (19)	178
C27—H27*C*⋯O2^iii^	0.98	2.52	3.2656 (19)	133

Symmetry codes: (i) 
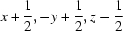
; (ii) 
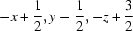
; (iii) 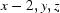.

**Fig. 1.**
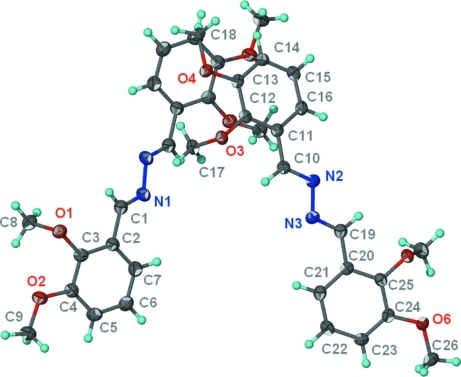
Thermal ellipsoid plot (Barbour, 2001) of the two molecules of C18H20N2O4 at the 70% probability level; hydrogen atoms are drawn as spheres of arbitrary radius.

## supplementary crystallographic information

### Comment

Several methoxy-substituted benzaldehyde azines have been reported, *e.g.*, 2,4-dimethyoxybenzaldehyde azine (Islam *et al.*, 2009). The compounds feature a C═N–N═C linkage that allows the two aromatic sytems to interaction. Such interaction results in a deep yellow coloration. In the reported compound, the rings are nearly co-planar. In the 2,3-dimethoxy analog (Scheme I), the rings are co-planar in the indpendent molecule lying on a center-of-inversion; in the other molecule, the rings are severely twisted (Fig. 1). Intermolecular weak C—H···O hydrogen bonding is present in the crystal strcture (Table 1).

### Experimental

2,3-Dimethyoxybenzaldehyde (1 g, 6 mmol) was dissolved in ethanol (15 ml) and to the solution was added hydrazine hydrate (0.3 ml, 6 mmol) followed by 5 drops of acetic acid. The mixture was heated for 3 h; slow evaporation of the solvent gave yellow crystals in 80% yield.

### Refinement

Carbon-bound H-atoms were placed in calculated positions [C—H 0.95 to 0.98 Å, *U*_iso_(H) 1.2 to 1.5*U*_eq_(C)] and were included in the refinement in the riding model approximation.

### Crystal data

**Table:**

C_18_H_20_N_2_O_4_	*F*(000) = 1044
*M**_r_* = 328.36	*D*_x_ = 1.305 Mg m^−^^3^
Monoclinic, *P*2_1_/*n*	Mo *K*α radiation, λ = 0.71073 Å
Hall symbol: -P 2yn	Cell parameters from 5647 reflections
*a* = 8.0294 (2) Å	θ = 2.3–29.3°
*b* = 17.9415 (5) Å	µ = 0.09 mm^−^^1^
*c* = 17.5258 (4) Å	*T* = 100 K
β = 96.660 (2)°	Prism, yellow
*V* = 2507.72 (11) Å^3^	0.30 × 0.25 × 0.20 mm
*Z* = 6	

### Data collection

**Table:**

Agilent SuperNova Dual diffractometer with an Atlas detector	5600 independent reflections
Radiation source: SuperNova (Mo) X-ray Source	4465 reflections with *I* > 2σ(*I*)
Mirror	*R*_int_ = 0.029
Detector resolution: 10.4041 pixels mm^-1^	θ_max_ = 27.5°, θ_min_ = 2.3°
ω scans	*h* = −10→10
Absorption correction: multi-scan (*CrysAlis PRO*; Agilent, 2010)	*k* = −18→22
*T*_min_ = 0.770, *T*_max_ = 1.000	*l* = −15→22
12722 measured reflections	

### Refinement

**Table:**

Refinement on *F*^2^	Primary atom site location: structure-invariant direct methods
Least-squares matrix: full	Secondary atom site location: difference Fourier map
*R*[*F*^2^ > 2σ(*F*^2^)] = 0.046	Hydrogen site location: inferred from neighbouring sites
*wR*(*F*^2^) = 0.125	H-atom parameters constrained
*S* = 1.03	*w* = 1/[σ^2^(*F*_o_^2^) + (0.0541*P*)^2^ + 1.0146*P*] where *P* = (*F*_o_^2^ + 2*F*_c_^2^)/3
5600 reflections	(Δ/σ)_max_ = 0.001
325 parameters	Δρ_max_ = 0.31 e Å^−^^3^
0 restraints	Δρ_min_ = −0.22 e Å^−^^3^

### Fractional atomic coordinates and isotropic or equivalent isotropic displacement parameters (Å^2^)

**Table:**

	*x*	*y*	*z*	*U*_iso_*/*U*_eq_	
O1	1.44670 (14)	0.12618 (6)	0.46827 (6)	0.0233 (3)	
O2	1.51150 (15)	0.22434 (7)	0.36049 (6)	0.0274 (3)	
O3	0.80577 (13)	0.08624 (6)	0.73656 (6)	0.0206 (2)	
O4	0.88407 (14)	−0.04039 (6)	0.81165 (6)	0.0229 (3)	
O5	−0.14383 (13)	0.28732 (6)	0.56994 (6)	0.0207 (3)	
O6	−0.24191 (14)	0.39718 (7)	0.47148 (6)	0.0270 (3)	
N1	0.99742 (18)	0.02682 (8)	0.47015 (7)	0.0244 (3)	
N2	0.33013 (17)	0.14903 (8)	0.66135 (7)	0.0230 (3)	
N3	0.32823 (17)	0.21485 (8)	0.61681 (7)	0.0219 (3)	
C1	1.1291 (2)	0.06789 (9)	0.47776 (9)	0.0227 (3)	
H1	1.2113	0.0607	0.5207	0.027*	
C2	1.1546 (2)	0.12573 (9)	0.42122 (9)	0.0216 (3)	
C3	1.3166 (2)	0.15129 (9)	0.41631 (8)	0.0210 (3)	
C4	1.3473 (2)	0.20464 (9)	0.36085 (9)	0.0225 (3)	
C5	1.2143 (2)	0.23323 (10)	0.31222 (9)	0.0270 (4)	
H5	1.2338	0.2696	0.2749	0.032*	
C6	1.0521 (2)	0.20851 (10)	0.31822 (10)	0.0284 (4)	
H6	0.9613	0.2289	0.2852	0.034*	
C7	1.0209 (2)	0.15493 (10)	0.37118 (9)	0.0263 (4)	
H7	0.9097	0.1379	0.3738	0.032*	
C8	1.5554 (2)	0.07435 (10)	0.43631 (10)	0.0290 (4)	
H8A	1.6445	0.0589	0.4761	0.044*	
H8B	1.6052	0.0982	0.3940	0.044*	
H8C	1.4907	0.0306	0.4169	0.044*	
C9	1.5503 (2)	0.27571 (11)	0.30281 (11)	0.0336 (4)	
H9A	1.6713	0.2852	0.3087	0.050*	
H9B	1.4902	0.3226	0.3083	0.050*	
H9C	1.5161	0.2545	0.2519	0.050*	
C10	0.4791 (2)	0.12549 (9)	0.68026 (8)	0.0200 (3)	
H10A	0.5707	0.1537	0.6659	0.024*	
C11	0.51268 (19)	0.05616 (9)	0.72347 (8)	0.0195 (3)	
C12	0.67881 (19)	0.03737 (9)	0.74863 (8)	0.0186 (3)	
C13	0.71782 (19)	−0.02919 (9)	0.78884 (8)	0.0194 (3)	
C14	0.5896 (2)	−0.07717 (9)	0.80322 (9)	0.0218 (3)	
H14A	0.6145	−0.1224	0.8303	0.026*	
C15	0.4237 (2)	−0.05849 (10)	0.77768 (9)	0.0235 (4)	
H15A	0.3363	−0.0916	0.7874	0.028*	
C16	0.3841 (2)	0.00711 (9)	0.73868 (9)	0.0222 (3)	
H16A	0.2704	0.0191	0.7222	0.027*	
C17	0.9006 (2)	0.06256 (10)	0.67612 (9)	0.0262 (4)	
H17A	0.9887	0.0991	0.6700	0.039*	
H17B	0.8259	0.0585	0.6279	0.039*	
H17C	0.9517	0.0139	0.6892	0.039*	
C18	0.9285 (2)	−0.10591 (10)	0.85626 (9)	0.0253 (4)	
H18A	1.0505	−0.1078	0.8691	0.038*	
H18B	0.8907	−0.1503	0.8265	0.038*	
H18C	0.8746	−0.1044	0.9036	0.038*	
C19	0.1780 (2)	0.23233 (9)	0.59042 (8)	0.0210 (3)	
H19A	0.0886	0.2030	0.6053	0.025*	
C20	0.1376 (2)	0.29503 (9)	0.53866 (8)	0.0193 (3)	
C21	0.2569 (2)	0.32947 (9)	0.49745 (8)	0.0216 (3)	
H21A	0.3706	0.3139	0.5048	0.026*	
C22	0.2081 (2)	0.38584 (10)	0.44645 (9)	0.0246 (4)	
H22A	0.2887	0.4084	0.4182	0.030*	
C23	0.0420 (2)	0.41041 (9)	0.43554 (9)	0.0234 (4)	
H23A	0.0103	0.4496	0.4004	0.028*	
C24	−0.0767 (2)	0.37737 (9)	0.47628 (9)	0.0209 (3)	
C25	−0.02836 (19)	0.31907 (9)	0.52731 (8)	0.0191 (3)	
C26	−0.2977 (2)	0.45460 (11)	0.41808 (11)	0.0309 (4)	
H26A	−0.4175	0.4637	0.4199	0.046*	
H26B	−0.2793	0.4390	0.3661	0.046*	
H26C	−0.2347	0.5004	0.4315	0.046*	
C27	−0.2600 (2)	0.23775 (10)	0.52664 (9)	0.0273 (4)	
H27A	−0.3380	0.2173	0.5603	0.041*	
H27B	−0.1981	0.1969	0.5057	0.041*	
H27C	−0.3231	0.2652	0.4844	0.041*	

### Atomic displacement parameters (Å^2^)

**Table:**

	*U*^11^	*U*^22^	*U*^33^	*U*^12^	*U*^13^	*U*^23^
O1	0.0249 (6)	0.0233 (6)	0.0214 (5)	0.0023 (5)	0.0017 (4)	0.0007 (4)
O2	0.0256 (6)	0.0275 (7)	0.0292 (6)	−0.0034 (5)	0.0027 (5)	0.0083 (5)
O3	0.0180 (6)	0.0206 (6)	0.0235 (5)	−0.0015 (5)	0.0043 (4)	−0.0011 (4)
O4	0.0182 (6)	0.0238 (6)	0.0262 (6)	0.0018 (5)	−0.0002 (4)	0.0054 (5)
O5	0.0182 (6)	0.0237 (6)	0.0205 (5)	−0.0017 (5)	0.0041 (4)	0.0006 (4)
O6	0.0200 (6)	0.0308 (7)	0.0307 (6)	0.0066 (5)	0.0051 (5)	0.0103 (5)
N1	0.0263 (8)	0.0247 (8)	0.0235 (7)	−0.0004 (6)	0.0086 (6)	0.0015 (6)
N2	0.0227 (7)	0.0229 (7)	0.0232 (7)	0.0017 (6)	0.0017 (5)	0.0029 (5)
N3	0.0223 (7)	0.0206 (7)	0.0225 (7)	0.0019 (6)	0.0012 (5)	0.0020 (5)
C1	0.0254 (9)	0.0215 (9)	0.0222 (8)	0.0028 (7)	0.0065 (6)	−0.0016 (6)
C2	0.0251 (9)	0.0188 (8)	0.0217 (8)	0.0015 (7)	0.0068 (6)	−0.0012 (6)
C3	0.0267 (9)	0.0175 (8)	0.0189 (7)	0.0014 (7)	0.0033 (6)	−0.0028 (6)
C4	0.0237 (9)	0.0213 (9)	0.0227 (8)	−0.0008 (7)	0.0041 (6)	−0.0019 (6)
C5	0.0312 (9)	0.0266 (9)	0.0234 (8)	0.0024 (8)	0.0045 (7)	0.0048 (7)
C6	0.0265 (9)	0.0305 (10)	0.0281 (8)	0.0053 (8)	0.0020 (7)	0.0035 (7)
C7	0.0249 (9)	0.0259 (9)	0.0289 (8)	0.0013 (7)	0.0059 (7)	0.0002 (7)
C8	0.0284 (9)	0.0244 (9)	0.0348 (9)	0.0052 (8)	0.0062 (7)	0.0016 (7)
C9	0.0312 (10)	0.0310 (10)	0.0397 (10)	−0.0009 (8)	0.0086 (8)	0.0133 (8)
C10	0.0196 (8)	0.0220 (8)	0.0182 (7)	−0.0002 (7)	0.0016 (6)	−0.0004 (6)
C11	0.0203 (8)	0.0216 (8)	0.0166 (7)	0.0014 (7)	0.0027 (6)	−0.0019 (6)
C12	0.0192 (8)	0.0196 (8)	0.0174 (7)	−0.0019 (6)	0.0035 (6)	−0.0031 (6)
C13	0.0182 (8)	0.0225 (8)	0.0173 (7)	0.0012 (7)	0.0013 (6)	−0.0026 (6)
C14	0.0249 (8)	0.0205 (8)	0.0203 (7)	0.0008 (7)	0.0038 (6)	0.0018 (6)
C15	0.0212 (8)	0.0258 (9)	0.0241 (8)	−0.0043 (7)	0.0048 (6)	−0.0002 (6)
C16	0.0169 (8)	0.0266 (9)	0.0230 (8)	0.0007 (7)	0.0025 (6)	0.0002 (6)
C17	0.0226 (9)	0.0284 (9)	0.0291 (9)	0.0017 (7)	0.0089 (7)	0.0018 (7)
C18	0.0227 (9)	0.0249 (9)	0.0276 (8)	0.0043 (7)	−0.0002 (6)	0.0064 (7)
C19	0.0206 (8)	0.0221 (9)	0.0203 (7)	0.0004 (7)	0.0030 (6)	−0.0021 (6)
C20	0.0212 (8)	0.0185 (8)	0.0176 (7)	0.0008 (7)	−0.0001 (6)	−0.0027 (6)
C21	0.0184 (8)	0.0244 (9)	0.0217 (8)	−0.0003 (7)	0.0011 (6)	−0.0026 (6)
C22	0.0235 (9)	0.0268 (9)	0.0241 (8)	−0.0041 (7)	0.0058 (6)	0.0004 (7)
C23	0.0247 (9)	0.0231 (9)	0.0221 (8)	0.0011 (7)	0.0019 (6)	0.0046 (6)
C24	0.0189 (8)	0.0228 (9)	0.0211 (7)	0.0021 (7)	0.0021 (6)	−0.0016 (6)
C25	0.0194 (8)	0.0208 (8)	0.0175 (7)	−0.0024 (7)	0.0036 (6)	−0.0020 (6)
C26	0.0245 (9)	0.0291 (10)	0.0385 (10)	0.0055 (8)	0.0012 (7)	0.0116 (8)
C27	0.0224 (9)	0.0302 (10)	0.0288 (9)	−0.0065 (8)	0.0013 (7)	0.0000 (7)

### Geometric parameters (Å, °)

**Table:**

O1—C3	1.3792 (19)	C10—C11	1.465 (2)
O1—C8	1.433 (2)	C10—H10A	0.9500
O2—C4	1.366 (2)	C11—C12	1.397 (2)
O2—C9	1.429 (2)	C11—C16	1.405 (2)
O3—C12	1.3794 (18)	C12—C13	1.403 (2)
O3—C17	1.4383 (19)	C13—C14	1.387 (2)
O4—C13	1.3632 (19)	C14—C15	1.396 (2)
O4—C18	1.4339 (19)	C14—H14A	0.9500
O5—C25	1.3796 (18)	C15—C16	1.380 (2)
O5—C27	1.440 (2)	C15—H15A	0.9500
O6—C24	1.3664 (19)	C16—H16A	0.9500
O6—C26	1.429 (2)	C17—H17A	0.9800
N1—C1	1.283 (2)	C17—H17B	0.9800
N1—N1^i^	1.419 (3)	C17—H17C	0.9800
N2—C10	1.276 (2)	C18—H18A	0.9800
N2—N3	1.4146 (19)	C18—H18B	0.9800
N3—C19	1.280 (2)	C18—H18C	0.9800
C1—C2	1.465 (2)	C19—C20	1.458 (2)
C1—H1	0.9500	C19—H19A	0.9500
C2—C3	1.392 (2)	C20—C25	1.393 (2)
C2—C7	1.406 (2)	C20—C21	1.407 (2)
C3—C4	1.406 (2)	C21—C22	1.376 (2)
C4—C5	1.385 (2)	C21—H21A	0.9500
C5—C6	1.391 (2)	C22—C23	1.396 (2)
C5—H5	0.9500	C22—H22A	0.9500
C6—C7	1.379 (2)	C23—C24	1.388 (2)
C6—H6	0.9500	C23—H23A	0.9500
C7—H7	0.9500	C24—C25	1.401 (2)
C8—H8A	0.9800	C26—H26A	0.9800
C8—H8B	0.9800	C26—H26B	0.9800
C8—H8C	0.9800	C26—H26C	0.9800
C9—H9A	0.9800	C27—H27A	0.9800
C9—H9B	0.9800	C27—H27B	0.9800
C9—H9C	0.9800	C27—H27C	0.9800
			
C3—O1—C8	113.74 (12)	C13—C14—C15	119.57 (15)
C4—O2—C9	117.40 (13)	C13—C14—H14A	120.2
C12—O3—C17	112.88 (12)	C15—C14—H14A	120.2
C13—O4—C18	117.00 (12)	C16—C15—C14	121.34 (15)
C25—O5—C27	113.87 (11)	C16—C15—H15A	119.3
C24—O6—C26	117.17 (13)	C14—C15—H15A	119.3
C1—N1—N1^i^	111.08 (16)	C15—C16—C11	119.75 (15)
C10—N2—N3	111.68 (13)	C15—C16—H16A	120.1
C19—N3—N2	110.69 (13)	C11—C16—H16A	120.1
N1—C1—C2	120.95 (15)	O3—C17—H17A	109.5
N1—C1—H1	119.5	O3—C17—H17B	109.5
C2—C1—H1	119.5	H17A—C17—H17B	109.5
C3—C2—C7	119.36 (15)	O3—C17—H17C	109.5
C3—C2—C1	118.68 (15)	H17A—C17—H17C	109.5
C7—C2—C1	121.96 (15)	H17B—C17—H17C	109.5
O1—C3—C2	119.57 (14)	O4—C18—H18A	109.5
O1—C3—C4	119.93 (15)	O4—C18—H18B	109.5
C2—C3—C4	120.45 (15)	H18A—C18—H18B	109.5
O2—C4—C5	125.23 (15)	O4—C18—H18C	109.5
O2—C4—C3	115.20 (14)	H18A—C18—H18C	109.5
C5—C4—C3	119.57 (16)	H18B—C18—H18C	109.5
C4—C5—C6	119.79 (16)	N3—C19—C20	123.12 (15)
C4—C5—H5	120.1	N3—C19—H19A	118.4
C6—C5—H5	120.1	C20—C19—H19A	118.4
C7—C6—C5	121.20 (16)	C25—C20—C21	119.17 (14)
C7—C6—H6	119.4	C25—C20—C19	117.91 (14)
C5—C6—H6	119.4	C21—C20—C19	122.85 (14)
C6—C7—C2	119.61 (16)	C22—C21—C20	119.77 (15)
C6—C7—H7	120.2	C22—C21—H21A	120.1
C2—C7—H7	120.2	C20—C21—H21A	120.1
O1—C8—H8A	109.5	C21—C22—C23	121.09 (15)
O1—C8—H8B	109.5	C21—C22—H22A	119.5
H8A—C8—H8B	109.5	C23—C22—H22A	119.5
O1—C8—H8C	109.5	C24—C23—C22	119.76 (15)
H8A—C8—H8C	109.5	C24—C23—H23A	120.1
H8B—C8—H8C	109.5	C22—C23—H23A	120.1
O2—C9—H9A	109.5	O6—C24—C23	125.21 (14)
O2—C9—H9B	109.5	O6—C24—C25	115.38 (14)
H9A—C9—H9B	109.5	C23—C24—C25	119.41 (15)
O2—C9—H9C	109.5	O5—C25—C20	119.13 (14)
H9A—C9—H9C	109.5	O5—C25—C24	120.00 (14)
H9B—C9—H9C	109.5	C20—C25—C24	120.79 (14)
N2—C10—C11	121.74 (15)	O6—C26—H26A	109.5
N2—C10—H10A	119.1	O6—C26—H26B	109.5
C11—C10—H10A	119.1	H26A—C26—H26B	109.5
C12—C11—C16	118.99 (15)	O6—C26—H26C	109.5
C12—C11—C10	118.67 (14)	H26A—C26—H26C	109.5
C16—C11—C10	122.32 (14)	H26B—C26—H26C	109.5
O3—C12—C11	119.60 (14)	O5—C27—H27A	109.5
O3—C12—C13	119.47 (14)	O5—C27—H27B	109.5
C11—C12—C13	120.88 (14)	H27A—C27—H27B	109.5
O4—C13—C14	125.27 (14)	O5—C27—H27C	109.5
O4—C13—C12	115.25 (14)	H27A—C27—H27C	109.5
C14—C13—C12	119.47 (14)	H27B—C27—H27C	109.5
			
C10—N2—N3—C19	171.89 (14)	C18—O4—C13—C12	176.95 (13)
N1^i^—N1—C1—C2	177.86 (15)	O3—C12—C13—O4	−1.4 (2)
N1—C1—C2—C3	−158.74 (15)	C11—C12—C13—O4	−178.69 (13)
N1—C1—C2—C7	20.1 (2)	O3—C12—C13—C14	177.86 (13)
C8—O1—C3—C2	106.62 (16)	C11—C12—C13—C14	0.5 (2)
C8—O1—C3—C4	−75.95 (18)	O4—C13—C14—C15	178.95 (14)
C7—C2—C3—O1	176.09 (14)	C12—C13—C14—C15	−0.2 (2)
C1—C2—C3—O1	−5.0 (2)	C13—C14—C15—C16	−0.4 (2)
C7—C2—C3—C4	−1.3 (2)	C14—C15—C16—C11	0.6 (2)
C1—C2—C3—C4	177.54 (14)	C12—C11—C16—C15	−0.2 (2)
C9—O2—C4—C5	−3.4 (2)	C10—C11—C16—C15	178.05 (14)
C9—O2—C4—C3	177.07 (15)	N2—N3—C19—C20	−176.19 (13)
O1—C3—C4—O2	3.9 (2)	N3—C19—C20—C25	−165.89 (15)
C2—C3—C4—O2	−178.72 (14)	N3—C19—C20—C21	17.2 (2)
O1—C3—C4—C5	−175.72 (14)	C25—C20—C21—C22	−0.4 (2)
C2—C3—C4—C5	1.7 (2)	C19—C20—C21—C22	176.53 (15)
O2—C4—C5—C6	179.92 (15)	C20—C21—C22—C23	0.9 (2)
C3—C4—C5—C6	−0.5 (2)	C21—C22—C23—C24	−0.4 (2)
C4—C5—C6—C7	−1.0 (3)	C26—O6—C24—C23	2.5 (2)
C5—C6—C7—C2	1.3 (3)	C26—O6—C24—C25	−177.83 (14)
C3—C2—C7—C6	−0.2 (2)	C22—C23—C24—O6	178.95 (15)
C1—C2—C7—C6	−179.01 (15)	C22—C23—C24—C25	−0.7 (2)
N3—N2—C10—C11	−177.09 (13)	C27—O5—C25—C20	−107.31 (16)
N2—C10—C11—C12	−173.93 (14)	C27—O5—C25—C24	75.94 (18)
N2—C10—C11—C16	7.8 (2)	C21—C20—C25—O5	−177.46 (13)
C17—O3—C12—C11	−105.52 (16)	C19—C20—C25—O5	5.5 (2)
C17—O3—C12—C13	77.10 (17)	C21—C20—C25—C24	−0.7 (2)
C16—C11—C12—O3	−177.65 (13)	C19—C20—C25—C24	−177.79 (14)
C10—C11—C12—O3	4.0 (2)	O6—C24—C25—O5	−1.7 (2)
C16—C11—C12—C13	−0.3 (2)	C23—C24—C25—O5	177.97 (14)
C10—C11—C12—C13	−178.66 (13)	O6—C24—C25—C20	−178.42 (13)
C18—O4—C13—C14	−2.2 (2)	C23—C24—C25—C20	1.3 (2)

Symmetry codes: (i) −*x*+2, −*y*, −*z*+1.

### Hydrogen-bond geometry (Å, °)

**Table:**

*D*—H···*A*	*D*—H	H···*A*	*D*···*A*	*D*—H···*A*
C9—H9B···O3^ii^	0.98	2.45	3.288 (2)	143
C14—H14A···O5^iii^	0.95	2.37	3.2890 (19)	161
C18—H18C···O6^iii^	0.98	2.54	3.5229 (19)	178
C27—H27C···O2^iv^	0.98	2.52	3.2656 (19)	133

Symmetry codes: (ii) *x*+1/2, −*y*+1/2, *z*−1/2; (iii) −*x*+1/2, *y*−1/2, −*z*+3/2; (iv) *x*−2, *y*, *z*.

## References

[bb1] Agilent (2010). *CrysAlis PRO* Agilent Technologies, Yarnton, England.

[bb2] Barbour, L. J. (2001). *J. Supramol. Chem.* **1**, 189–191.

[bb3] Islam, M. A. A. A. A., Tarafder, M. T. H., Alam, M. A., Guidolin, N. & Zangrando, E. (2009). *Acta Cryst.* E**65**, o2560. 10.1107/S1600536809038082 PMC2970277 21578000

[bb4] Sheldrick, G. M. (2008). *Acta Cryst.* A**64**, 112–122. 10.1107/S0108767307043930 18156677

[bb5] Westrip, S. P. (2010). *J. Appl. Cryst.* **43**, 920–925.

